# Exploring perceived challenges, adoption, and assessment of Western values of democracy and human rights in Palestine in the 2023 War on Gaza

**DOI:** 10.1038/s41598-024-60147-x

**Published:** 2024-06-14

**Authors:** Oqab Jabali, Heba Sleem, Abed Alkarim Ayyoub, Munther Saeedi, Yousef Alawneh, Muath Ishtaiyeh

**Affiliations:** 1 Language Center, Faculty of Humanities and Education Sciences, An-Najah National University, Nablus, Palestine; 2 Department of Teaching Methods, Faculty of Humanities and Education Sciences, An-Najah National University, Nablus, Palestine; 3 Faculty of Humanities and Education Sciences, An-Najah National University, Nablus, Palestine; 4 Islamic University of Minnesota, Minnetonka, USA; 5 Istiqlal University, Jericho, Palestine

**Keywords:** Adoption/assessment of Western values, Democracy, Human rights, 2023 War on Gaza, Perceived challenges, Psychology, Environmental social sciences

## Abstract

This study delved into the dynamics of perceived challenges, adoption, and assessment of Western values of democracy and human rights among university students in Palestine, particularly in the aftermath of the 2023 War on Gaza. A mixed-methods strategy was used in the research, with a participant pool of 384 students representing a range of demographics. By exploring the impact of geopolitical events, the results revealed a positive link between perceived challenges and the assessment of Western values. Although there is a notable gender and geographic difference in the assessment and adoption of Western values, females and those living in cities and villages are shown to have greater perceived challenges with these values. The qualitative component, including interviews with 12 students, provided valuable insights into the postwar evolution of Palestinian perspectives, highlighting a notable shift in attitudes, initially characterized by belief in the superiority of Western values, followed by a decline in faith during the war. This decline is attributed to traumatic events, biased media narratives, and the contradiction between idealized standards and harsh realities. In conclusion, the study emphasizes the need for a comprehensive understanding of the multifaceted influences on Palestinian perceptions of Western values.

## Introduction

Palestinians view Western democracy and human rights with skepticism due to perceived double standards and lack of action regarding the Israeli occupation^1^. The 2023 war on Gaza intensified their disillusionment with humanitarian organizations and Western governments, leading to high levels of cynicism towards human rights and democracy^2^. Despite numerous UN resolutions and reports from human rights organizations condemning Israeli actions, Western governments have failed to impose sanctions on Israel, further eroding Palestinian trust in Western principles of democracy and human rights^3^. Palestinians' skepticism towards Western democracy and human rights is deeply rooted in their experiences and observations of official international responses to the Israeli occupation^4^. Palestinians noted a pronounced lack of equitable moral and political outrage when comparing the attention given to their plight with other global conflicts. This discrepancy has intensified feelings of betrayal and disillusionment towards Western governments, whom they perceive as upholding double standards^5^.

The skepticism is not just about the abstract concepts of democracy and human rights but also about the entities that promote these values^6^. Many Palestinians are disillusioned with western governments and international humanitarian organizations, which they see as ineffective due to their reliance on funding from governments that remain passive or biased in dealing with the Israeli occupation. This sentiment, as reflected in a public opinion study^4^, underscores the skepticism among some members of the Palestinian public. This disillusionment is compounded by daily exposures to the harsh realities of the occupation, reinforcing a perception that human rights are selectively applied and that Palestinian rights are consistently marginalized^7^.

Western governments have been criticized for exhibiting double standards, complicit silence, and refusal to respond to calls for a cessation of hostilities against Palestinians^8^. Despite their support for Ukraine, it is widely believed that Western leaders have not responded to calls for a ceasefire against Palestinians in a timely manner^1^. They have endorsed Israel's right to self-defense, authorizing genocide and extermination^9^. The United States has not only neglected to advocate for a humanitarian ceasefire but also thwarted four UNSC draft resolutions^10^. They have provided material and logistical support, moved aircraft carriers and naval ships to the eastern Mediterranean, and inaugurated an air bridge between Washington and Tel Aviv to help Israel^11^. On the other hand, they have undertaken measures to support the Palestinian people, such as the provision of a floating dock for aid deliveries and other humanitarian assistance. Nevertheless, the complexity and geopolitical factors of conflicts dictate their course of action^11^.

The Israeli army obstructs survivors' access to basic needs, including food, water, and medical care^12^. This underscores our argument that the Western-embraced human rights system is morally bankrupt and operates on selectively applied double standards that undermine its effectiveness. While many Palestinians generally hold a positive view of the EU's peacebuilding model^13^, there is significant disappointment with the EU's foreign policy behavior^14^.

**Figure 1:**
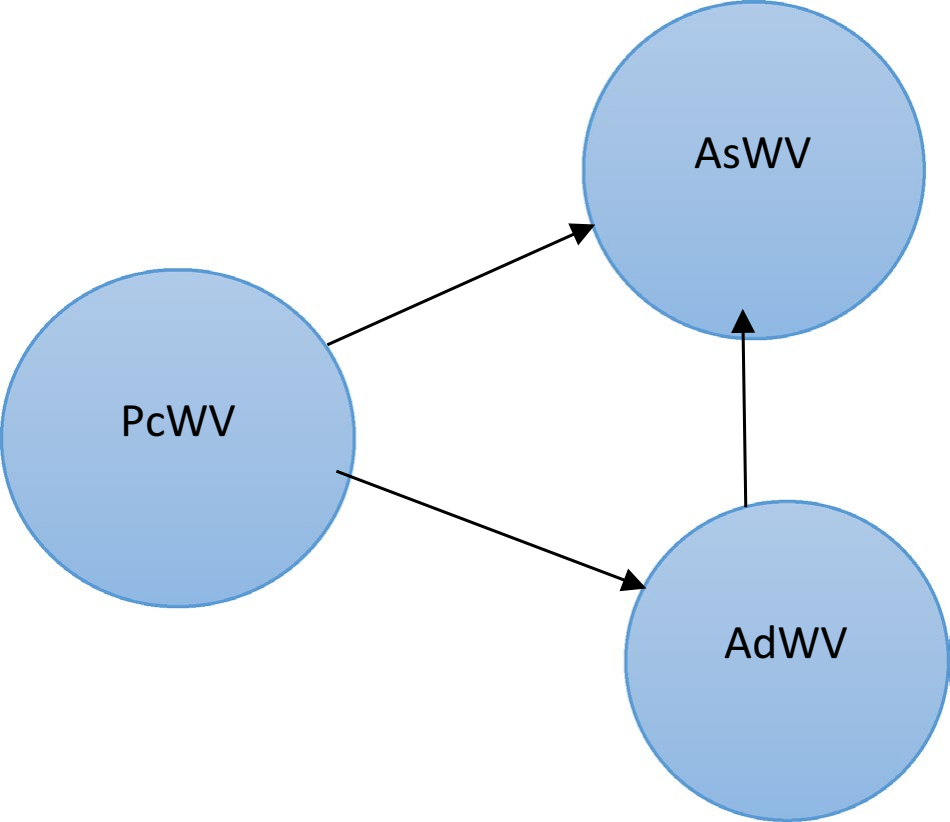
Hypothesized study model. *AsWV* assessment of Western values, *AdWV* adoption of Western values, *PcWV* perceived challenges of Western values.

The researchers’ main objective is to explore the complex dynamics surrounding Palestinian university students' perceptions of the perceived challenges, adoption trends, and assessment mechanisms associated with Western concepts of democracy and human rights. This exploration is particularly important in the context of the Gaza War of 2023 to provide a more refined grasp of how this geopolitical event affects people’s perspectives and reactions to Western values. This research aims to provide important insights into the complex relationship between perceived challenges, the adoption of values, and the subsequent assessments made by university students in Palestine against the backdrop of a momentous geopolitical event by examining these interrelated aspects. The researchers proposed a special model, as shown in Fig. 1, to answer the following questions.

### Research questions

- Q1: How do the challenges pertaining to Western ideals of democracy and human rights impact Palestinian university students' evaluation of these values?
- Q2: What effect do Western democracy and human rights adoption have on how Palestinian university students perceive these ideals?
- Q3: How does the adoption of Western values of Democracy and Human Rights moderate the relationship between challenges and the assessment of these values among university students in Palestine?

### Research hypotheses

- H1: There is a positive correlation between the challenges arising from the Western values of Democracy and Human Rights and the assessment of these values among university students in Palestine. The more challenges there are, the greater the inclination toward assessing the values.
- H2: There is a negative correlation between the adoption of Western values of Democracy and Human Rights and the assessment of these values among university students in Palestine. The greater the adoption is, the less the inclination to assess the values.
- H3: There is a negative correlation between the challenges arising from the Western values of Democracy and Human Rights and the assessment of these values among university students in Palestine, with the adoption of these values potentially moderating this correlation.

**To complement the quantitative data, the researchers conducted structured interviews with some participants to answer the following questions:**

1. Prior to the 2023 War on Gaza (Battle of the Al-Aqsa Flood), how much did you trust in Western values?
2. Did you notice a decline in the admiration for Western values among your family members and friends after the 2023 War on Gaza?
3. Do you think it is obvious that Western values and people's values and beliefs are at odds with one other?
4. Do you continue to draw from the opinions of Western thinkers and influencers who advocate democracy and human rights?

## Methods

To thoroughly examine the complex dynamics within this particular sociopolitical setting, this study employs a mixed-method approach. This study aims to offer a comprehensive understanding of the difficulties encountered, the degrees of adoption, and the evaluation of Western values of democracy and human rights among Palestinian university students, especially in the wake of the 2023 War on Gaza. It does this by integrating both quantitative and qualitative methodologies. To enable a more comprehensive interpretation of the study's findings, this comprehensive method attempts to capture the multidimensional aspect of the phenomenon under examination.

### Participants

The surveys being openly accessible via Google Drive suggested that participants likely voluntarily engaged in convenience sampling. The study included a sample of 384 university students in Palestine, reflecting a broad demographic spectrum, as detailed in Table 1. Notably, the gender distribution revealed that a majority of the participants were female, constituting 70% of the sample, while 30% were male. This gender composition underscores the importance of considering gender dynamics in the analysis of attitudes toward Western values of democracy and human rights.

**Table 1:** Demographic characteristics.

Variable	Level	Count	%
Gender	Male	115	0.30
	Female	269	0.70
Faculty	Scientific	130	0.34
	Humanities	216	0.56
	Graduate	38	0.10
Level of education	First-year	73	0.19
	Second year	86	0.22
	Third year	93	0.24
	Fourth year or above	132	0.34
Place of residence	City	145	0.38
	Village	219	0.57
	Camp	20	0.05
Total		384	1.00

In terms of academic background, the sample exhibited diversity, with 34% belonging to scientific faculties, 56% to humanities faculties, and 10% enrolled in graduate studies. This variation in academic disciplines suggests that the study could capture a range of perspectives and insights from students across different fields of study.

Furthermore, the distribution of participants according to their living environment was stratified, with 38% residing in cities, 57% in rural areas, and 5% in camp surroundings. This geographic diversity may reflect differences in socioeconomic backgrounds, access to resources, and exposure to various societal norms and values. Such stratification enables a more nuanced understanding of how geographical contexts might influence attitudes toward Western values of democracy and human rights among Palestinian university students.

Qualitatively, we employed snowball sampling to ensure representation across all demographic variables, fostering participant diversity. Initially, when interviewing a larger cohort of students, we deliberately narrowed the final sample to 12 to mitigate data redundancy and ensure comprehensive insights.

Given that only one university was chosen for this study, it is important to note that it was selected for specific reasons. This university stands out as the largest and most established institution in Palestine and is renowned for its prestige and long-standing reputation. Palestinian students from various parts of the West Bank and areas within the Green Line are drawn to this university due to its esteemed Graduate School, as well as its faculties of Engineering and Medicine, which are particularly influential in attracting students' attention. Therefore, while the study's focus was on a single university, its prominence and broad appeal within the Palestinian academic landscape contribute to the representativeness of the findings among university students in the West Bank.

### Statistical analysis

The statistical analysis employed in this study involved exploratory factor analysis (EFA) to identify underlying structures in respondents' opinions of Western ideals. EFA utilized parallel analysis with oblique promax rotation and main component extraction, executed in JASP version 18.3. Significant correlations between items and factors were determined by loadings exceeding 0.4, leading to the identification of 12 items categorized into three primary domains (Assessment of Western values, Adoption of Western values, and Perceived Challenges of Western values), explaining 73% of the observed variation.

Subsequently, structural equation modeling (SEM), following^15^ approach, was applied to thoroughly examine the measurement and structural models, ensuring questionnaire validity and reliability. CFA initially developed the measurement model, followed by SEM to scrutinize causal links across constructs and test the structural model. These analyses were conducted using JASP 18:3, AMOS 24, and SPSS 26.

Validity checks included standardized regression weight (SRW), composite reliability (C.R.), Cronbach's α, and average variance extracted (AVE) values, all surpassing acceptable thresholds. Discriminant validity was confirmed based on^16^ methods.

Structural model evaluation adhered to defined criteria encompassing the Tucker‒Lewis index (TLI), incremental fix index (IFI), adjusted GFI (AGFI), comparative fit index (CFI), root mean square error of approximation (RMSEA), χ^2^/df ratio, and significance of the chi-square statistic (χ^2^). The model met these criteria, with GFI, AGFI, IFI, and CFI exceeding 0.9, RMSEA below 0.08, and χ^2^/df ratio below 3, validating its appropriateness for capturing the data's fundamental structure.

The study analyzed the causal paths in the structural model using standardized path coefficients and variance explained (R2). The path diagram provided insights into the strength and direction of relationships between variables. The researchers used a four-way ANOVA to determine the impact of demographic variables (gender, faculty, academic level, and place of residence) on the AdWV, AsWV, and PcWV.

### Study tools

Qualitatively, the researchers conducted semi-structured interviews with 12 Palestinian students to understand their perceptions of Western values such as democracy and human rights. The interviews were conducted in Arabic, allowing for freedom of expression and communication. This study aimed to gather diverse perspectives on Palestinian perceptions of Western values, considering all genders, academic levels, and years of study.

To gather quantitative data, an Arabic-language self-administered questionnaire was given to the participants. The questionnaire was divided into two sections. First, demographic details, including gender, faculty, level of education and place of residence, were collected. The second section comprised twenty-nine items designed to elicit students' perspectives on democracy and human rights as Western ideals. The respondents expressed their agreement with these items on a five-point Likert scale, with response options ranging from “Strongly Agree” (1) to “Strongly Disagree” (5).

To condense the data into a more comprehensible collection of summary variables, the researchers used exploratory factor analysis (EFA) to identify underlying structures in respondents' opinions of Western ideals. Parallel analysis with oblique promax rotation and main component extraction were used in the EFA, which was carried out with JASP version 18.3. The identification of significant correlations between items and factors was contingent upon loadings higher than 0.4. The analysis identified 12 items categorized into three primary domains (Assessment of Western values, Adoption of Western values and Perceived Challenges of Western values), explaining 73% of the observed variation, as shown in Table 2.

**Table 2:** Loading factors of items on the constructs from EFA analysis.

Factors	Assessment of Western values	Adoption of Western values	Navigating challenges	Variance
Q18	0.902			0.32
Q17	0.889			
Q11	0.848			
Q10	0.806			
Q16	0.757			
Q7		0.901		0.21
Q5		0.81		
Q8		0.802		
Q6		0.769		
Q27			0.921	0.2
Q26			0.851	
Q25			0.831	
Q24				
Total				0.73

The Assessment of Western values factor, which consists of five items with loading factors ranging from 0.757 to 0.902, significantly contributes to 32% of the dataset's variance, indicating a strong association between the items and the construct. The adoption of the Western values factor, which consists of four items with loading factors ranging from − 0.769 to − 0.901, significantly contributes to 21% of the total variance in the dataset, highlighting its unique inverse relationship. The Perceived Challenges of Western values factor, consisting of three items with loading factors ranging from 0.831 to 0.921, explains 20% of the dataset's variance, indicating a strong positive relationship between the items and the construct.

### Structural modeling analysis

The structural equation modeling (SEM) technique created by^16^ was applied to facilitate a thorough examination of the measurement and structural models, thereby guaranteeing the validity and reliability of the questionnaire. CFA was first used in the development of the measurement model. Second, SEM was employed to examine the causal links across each construct and test the structural model of the research concept. These measures were carried out using the statistical analysis software packages JASP 18:3, AMOS 24, and SPSS 26.

The standardized regression weight (SRW), composite reliability (CR), Cronbach's α, and AVE values were all above the minimally acceptable values, which are 0.7, 0.5, 0.7, and 0.5, respectively. These statistics imply a high degree of reliability and the production of very consistent results. Consequently, the findings verified that each construct has an adequate level of convergent validity. The results in Table 3 demonstrated that the SRW, CR, and AVE all exceeded their respective thresholds and that the CR for each construct was greater than the AVE.

**Table 3:** Reliability coefficients.

Item	Estimate	P	SRW	Cronbach's α	CR	AVE
Assessment of Western values						
Q18	0.952	< 0.001	0.882	0.92	0.93	0.81
Q17	0.91	< 0.001	0.859			
Q11	1		0.838			
Q10	0.984	< 0.001	0.786			
Q16	0.921	< 0.001	0.767			
Adoption of Western values						
Q7	0.882	< 0.001	0.895	0.83	0.89	0.84
Q5	0.92	< 0.001	0.8			
Q8	1		0.772			
Q6	0.713	< 0.001	0.769			
Perceived challenges of Western values						
Q27	0.929	< 0.001	0.901	0.9	0.95	0.88
Q26	1		0.831			
Q25	0.912	< 0.001	0.823			

Then, employing the methods suggested by^17^, the results were examined for discriminant validity. Because the square root of the AVE was greater than the correlation for every pair of constructs, the results indicated that each construct was distinct. Thus, discriminant validity is established as shown in Table 4.

**Table 4:** Discriminant validity.

	M	SD	Adoption of Western values	Perceived challenges of Western values	Assessment of Western values
Adoption of Western values	3.11	0.81	**0.92**		
Perceived challenges of Western values	4.08	0.99	− 0.48	**0.94**	
Assessment of Western values	1.3	0.66	− 0.57	0.46	**0.90**

Significant values are given in bold.

Structural equation modeling is customarily used to evaluate structural models based on defined criteria. The criteria included the Tucker‒Lewis index (TLI), incremental fix index (IFI), adjusted GFI (AGFI), comparative fit index (CFI), RMSEA, χ^2^/df ratio, and significance level of the chi-square statistic (χ^2^). These criteria act as indicators for assessing the goodness of fit of the model. A detailed analysis of the proposed model against these criteria is provided in Table 5, which shows that all goodness-of-fit indices are within the designated ranges. In particular, the GFI, AGFI, IFI, and CFI all exceeded the suggested 0.9 level, and the RMSEA was less than 0.08. Moreover, Table 5 shows that the χ^2^/df ratio is smaller than 3. This adherence to the predetermined standards validates the model's suitability for capturing the fundamental structure of the data and bolsters its validity within the study's parameters.

**Table 5:** Model fit indices.

Index	Value
Chi square (χ^2^)	χ^2^ (51) = 135.46, P = 0.00
χ^2^/df	2.65
Comparative fit index (CFI)	0.95
T-size CFI	0.926
Tucker‒Lewis index (TLI)	0.935
Bentler–Bonett nonnormed fit index (NNFI)	0.935
Bentler–Bonett normed fit index (NFI)	0.935
Bollen's relative fit index (RFI)	0.915
Bollen's incremental fit index (IFI)	0.95
Relative noncentrality index (RNI)	0.95
Root mean square error of approximation	0.068

### Ethics approval and consent to participate

The research was approved by the ethics committee of the Deanship of Scientific Research, the dean of the Faculty of Humanities, the director of the language Centre, and the IRB at the university. Oral informed consent was obtained from the participants. The endorsement by the An-Najah Institutional Review Board (IRB) for informed consent and the oral informed consent procedure confirmed the study's compliance with the ethical principles delineated in the 1964 Declaration of Helsinki.

## Quantitative results

Hypothesis testing: Properties of the causal paths, including standardized path coefficients and variance-explained R^2^.

**Figure 2:**
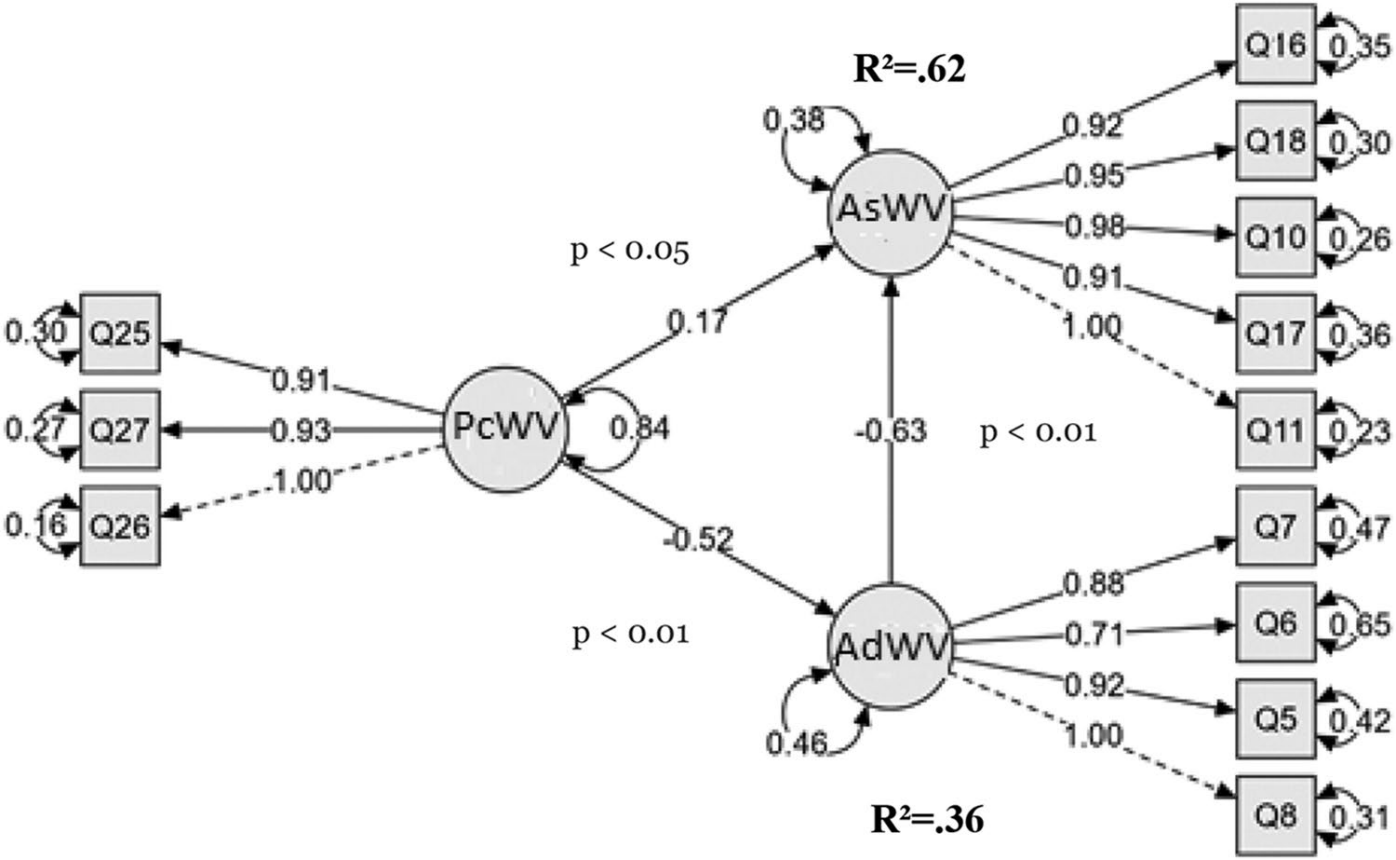
Model inner and outer variables.

After a thorough investigation of causal path attributes using variance explained (R2) and standardized path coefficients during the hypothesis testing stage, the subsequent analysis focused on the model's inner variables, as shown in Fig. 2.

The developed hypotheses are strongly supported by the structural model analysis results. First, the substantial influence of Perceived Challenges of Western values (PcWV) on both the Assessment of Western values (AsWV) and Adoption of Western values (AdWV) supports Hypothesis 1, which states that there is a positive correlation between challenges pertaining to Western values of Democracy and Human Rights and the assessment of these values among Palestinian university students. A greater tendency to evaluate these values is correlated with perceived challenges, as confirmed by the positive standardized coefficient (β = 0.17, p < 0.05).

Second, the substantial negative influence of the PcWV on the AdWV (β = − 0.63, p < 0.01) supports Hypothesis 2, which postulates a negative link between students' adoption of Western values and their assessment. This suggests that greater adoption rates are associated with a decreased tendency to evaluate Western values. Finally, the substantial negative effect of PcWV on AdWV (β = − 0.52, p < 0.01) supports Hypothesis 3, which postulates a negative correlation between challenges and assessment moderated by the adoption of values. This suggests that adoption reduces the negative correlation between challenges and assessment. The robustness of these results is shown by the overall significance of all item estimates at p = 0.001, which offers an extensive understanding of the interaction between challenges to, adoption of, and assessment of Western values among Palestinian university students. Table 6 provides an extensive representation of the direct, indirect, and cumulative effects of each construct on other constructs, demonstrating the importance of all item values at p = 0.001 for the outer variables.

**Table 6:** Direct and indirect effects.

Effect		Estimate	z value	P
Direct	PcWV → AsWV	− 0.27	− 7.126	< 0.001
Indirect	PcWV → AdWV → AsWV	− 0.25	− 8.131	< 0.001
Total		− 0.52	− 5.231	< 0.001

To determine the effects of demographic variables (gender, faculty, academic level, and place of residence) on the AdWV, AsWV and PcWV, the researchers carried out a four-way ANOVA; the results are shown in Table 7.

**Table 7:** Four-way ANOVA.

Dependent variable	Source	SS	Df	MS	F	P	LSD
AdWV	Gender	0.242	1	0.242	0.374	> 0.05	
	Faculty	1.938	2	0.969	1.498	> 0.05	
	Level of education	2.347	3	0.782	1.209	> 0.05	
	Place of residence	1.618	2	0.809	1.251	> 0.05	
	Total	254.35	383				
AsWV	Gender	0.155	1	0.155	0.339	> 0.05	
	Faculty	0.266	2	0.133	0.292	> 0.05	
	Level of education	0.111	3	0.037	0.082	> 0.05	
	Place of residence	0.264	2	0.132	0.29	> 0.05	
	Total	167.13	383				
PcWV	Gender	5.45	1	5.45	6.299	< 0.05	F > M
	Faculty	0.679	2	0.339	0.392	> 0.05	
	Level of education	2.195	3	0.732	0.845	> 0.05	
	Place of residence	5.327	2	2.664	3.078	< 0.05	C&V > C
	Total	372.28	383				

*F* female, *M* male, *C* city, *V* village.

The demographic variables had no significant effects on the AsWV or the AdWV. Nevertheless, PcWV revealed a noteworthy variation between males and females, with a significant sex effect (p < 0.05). While there are no significant effects for faculty, level of education, or place of residence (p > 0.05), the LSD post hoc test reveals that for the gender category, women have higher mean scores than men, and for place, city and village scores are higher than those of the camp.

## Qualitative results

Qualitatively, we employed snowball sampling to ensure representation across all demographic variables, fostering participant diversity. Initially, when interviewing a larger cohort of students, we deliberately narrowed the final sample to 12 to mitigate data redundancy and ensure comprehensive insights. Among the 12 students included in our study, there was an equal split in gender, with 6 identified as male and 6 as female. In terms of academic backgrounds, 5 students were from the Scientific faculty, 4 were from the Humanities faculty, and 3 were graduate students. Regarding their educational levels, there were 5 first-year, 3 second-year, 2 third-year, and 2 fourth-year or above students. In terms of residence, 4 students lived in urban areas, 6 in rural villages, and 2 in camp environments. This diverse mix of participants provides a broad representation across various demographics, ensuring a comprehensive perspective in our study.

Subsequent to the quantitative data analysis and result extraction, the researchers developed four open-ended questions to fully capture the viewpoints of the participants (12) male and female students who answered the study survey.

To identify important themes while allowing for interpretation, the researchers chose to employ thematic analysis, which entails looking for similar patterns throughout the dataset and interpreting them^16^.

**Question One: Prior to the 2023 War on Gaza (battle of the Al-Aqsa flood), how much did you trust in Western values?**

The question about the extent of belief in Western values before the 2023 War on Gaza yielded divergent responses. Students acknowledged a significant influence, with a perception of Western values as inherently superior, representing a more commendable model than their own values and cultural ethos.

"Following the conflict, we witnessed a notable shift in their status from leaders to individuals grappling with feelings of inadequacy and vulnerability." (Student 1)

Postwar, our perspective on them transitioned from being esteemed figures to ones struggling with a newfound sense of inferiority and marginalization." (Student 3)

"After the war, our view of them changed from being at the forefront to feeling overshadowed and undervalued, reflecting a significant reversal in their perceived status and prestige." (Student 4)

"In the aftermath of the war, our perception of their once dominant role evolved into one marked by a sense of diminished importance and diminished influence." (Student 2)

However, following the aforementioned conflict, a pronounced shift in perspective was observed, leading to a substantial decline in faith in Western values. In contrast, respondents S5, S6, and S12 maintained a steadfast position and affirmed that they had not initially believed in Western principles.

"We have never believed in Western values from the outset. Our principles have consistently differed from those promoted by the West." (Student 5)

"Our lack of trust in Western values has been there from the beginning. We have always seen them as incompatible with our own beliefs." (Student 6)

"From the start, we have had no faith in Western values. Our cultural background has shaped our perspective, leading us to view them with skepticism." (Student 12)

In addition, respondents S2 and S11 also described a changed perspective after the war, highlighting a greater dependence on their beliefs and values of religion. Student 2 argued that "After the war, I leaned more on my religious beliefs for guidance and strength." On the other hand, Student 11 stated, "The war made me prioritize my religious values for stability and purpose." In the context of international norms, participants (S3, S12) agreed that a double standard exists:

"Many argue the West employs a double standard regarding Palestine—advocating for human rights elsewhere while overlooking violations in the region." (Student 3)

"The West faces criticism for a perceived double standard regarding Palestine—championing democracy and rights but maintaining policies seen as biased against Palestinians." (Student 12)

**Question Two: Did you notice a decline in the admiration for Western values among your family members and friends after the 2023 War on Gaza?**

All participants agreed that there was a significant reduction in the appreciation of Western values among their families and friends. Some students argued that:

"Yes, some family and friends lost admiration for Western values post-2023 Gaza War due to perceived bias." (Student 3)

"No, admiration for Western values remained consistent despite the 2023 Gaza War." (Student 1)

"Mixed reactions occurred post-2023 Gaza War: some questioned Western values, while others upheld them." (Student 8)

"Post-2023 Gaza War, views on Western values varied: some questioned integrity, others emphasized importance." (Student 6)

"The 2023 Gaza War sparked debates on Western values; some lost faith, others upheld principles." (Student 5)

"Post-2023 Gaza War, some soured on Western values, citing perceived hypocrisy among family and friends." (Student 9)

"Opinions on Western values split post-2023 Gaza War: some felt disillusioned, while others remained steadfast in their support among family and friends." (Student 11)

Furthermore, other students expressed the viewpoint that the West advocates for freedoms and human rights in theory but does not effectively implement them in reality. Individual respondents believe the following:

"The West governments talk a lot about freedoms and human rights, but their support for regimes with poor records says otherwise." (Student 2)

"Many Western governments prioritize politics over human rights, especially when it benefits them economically." (Student 4)

"The West's actions contradict their talk about freedoms and human rights, especially in regions they're involved in." (Student 7)

Participants (10 and 12) additionally asserted that Western values are exclusive to the West, as shown here: "Western values are inherently confined to Western societies," remarked (Student 10). Another participant (Student 12) argued that "This viewpoint challenges the assumption of the universal applicability of Western values beyond Western borders." One participant reported no decline in the glorification of Western values within their environment, arguing that "In my observation, the glorification of Western values within our community remains consistent” (Student 11).

**Question Three: "Do you think it is obvious that Western values and our people's values and beliefs are at odds with one other?**

Most participants agreed that there is a significant contradiction between Arab-Islamic principles and Western values. Furthermore, individuals S1, S2, S3, and S9 acknowledged a heightened consciousness of this inconsistency, spurring a more resolute dedication to Arab and Islamic values.

"Yes, it is evident that Arab-Islamic principles often clash with Western values. Many participants highlighted this contradiction, leading to a stronger commitment to our own cultural and religious values." (Student 2)

"Absolutely, there's a significant disparity between Arab-Islamic principles and Western values, as acknowledged by multiple participants. This awareness has prompted some to reaffirm their dedication to our cultural heritage and beliefs." (Student 3)

"The contradiction between Arab-Islamic principles and Western values is clear, as noted by eleven participants. This recognition has fueled a renewed emphasis on Arab and Islamic values among some, while others believe Western values pose a threat to our communities." (Student 9)

"Yes, there's a notable contradiction between Arab-Islamic principles and Western values, as emphasized by multiple participants. This realization has led to a deeper commitment to our own values for some, while others see Western values as irrelevant or even harmful." (Student 1)

On the other hand, participants (S6, S8, and S10) agreed that the West attempted to impose values that endangered our communities:

Student 6 "warned against the dangers of the West imposing values that could harm our communities."

Participant S8 "expressed concerns about the West's attempts to enforce values seen as risky for our communities."

Finally, Student 10 "cautioned against the imposition of Western values, advocating for the preservation of our community's well-being."

However, some other participants (S1, S2 and S11) expressed the opinion that Western values are unimportant. They elaborated on their belief that Western values hold little importance within our community, emphasizing the need to prioritize our own cultural norms and beliefs over those imposed from external sources.

**Question four: Do you continue to be drawn to the opinions of Western thinkers and influencers who advocate for democracy and human rights?**

Participants (1, 4, 5, 6, and 10) all agreed that they were no longer drawn to any Western intellectuals or influencers who promoted the Western version of democracy and human rights. They cited the following justifications:

"I found Western intellectuals and influencers advocating for democracy and human rights less appealing due to perceived hypocrisy or inconsistency in their promotion of these values." (Student 6)

"I was disillusioned with Western intellectuals and influencers promoting democracy and human rights because their narratives are detached from the realities and complexities of non-Western societies." (Student 4)

"I became disenchanted with Western intellectuals and influencers advocating for democracy and human rights; their messages are culturally insensitive." (Student 10)

"We were no longer attracted to Western intellectuals and influencers championing democracy and human rights, as their narratives failed to address the systemic inequalities and injustices perpetuated by Western governments." (Student 1)

"I tried to distance myself from Western intellectuals and influencers promoting Western democracy and human rights because their narratives are Eurocentric and failing to acknowledge the diverse perspectives and approaches to governance present in Palestine." (Student 5)

In addition, participants (2, 3, 4, 5, 6, 9, 11, and 12) agreed that they were initially attracted to influential figures but decided not to follow them on social media after the Gaza War for two reasons: (1) “due to perceived biases or inadequate responses to the Palestinian-Israeli conflict” and (2) because “most of them failed to address or adequately respond to the humanitarian crisis in Gaza."

The responses provided by participants in the survey revealed several interconnected themes regarding their perceptions of Western values and influences, particularly in the context of the 2023 War on Gaza.

Initially, participants expressed varying degrees of trust and admiration for Western values. Some viewed Western values as inherently superior, representing a commendable model that overshadowed their own cultural ethos. However, the conflict in Gaza marked a turning point, leading to a notable shift in perspective. Participants described feeling disillusioned with Western values, citing perceived hypocrisy and inconsistency in their application, particularly concerning the Palestinian-Israeli conflict.

This disillusionment extended beyond individual perceptions to encompass broader societal attitudes. Participants observed a significant decline in the appreciation of Western values among their families and friends following the Gaza War, attributing this shift to perceived bias or inadequacy in Western responses to the conflict. Moreover, participants expressed a growing disinterest in following Western intellectuals or influencers advocating for Western democracy and human rights. They cited reasons such as perceived detachment from non-Western realities, cultural insensitivity, or Eurocentrism, leading to a rejection of Western narratives in favor of alternative viewpoints.

In contrast, some participants reported a heightened reliance on their religious beliefs for stability and purpose in the aftermath of the war, indicating a shift away from Western values toward religious values. Furthermore, participants recognized a significant contradiction between Arab-Islamic principles and Western values, leading to a reaffirmation of commitment to Arab and Islamic values. They also expressed concerns about attempts by the West to impose values perceived as harmful or incompatible with their communities, advocating for the preservation of their cultural heritage and well-being.

## Discussion

Quantitatively, the study revealed a positive correlation between perceived challenges of Western values and the assessment of these values among Palestinian university students. This is supported by the structural model analysis results. However, the study showed a negative link between students' adoption of Western values and their assessment, suggesting that greater adoption rates decrease the tendency to evaluate these values.

The first finding of the study is that Palestinian university students' assessments of Western ideals and their reported difficulties with them are positively correlated. This result is consistent with previous research, which highlights how geopolitical events—such as the Gaza War of 2023—have an impact on how people perceive and assess Western ideals^4,18^.

On the other hand, the second finding reveals a negative correlation between students' assessment and their adoption of Western values, indicating that greater adoption rates reduce the propensity to appraise these values. This result aligns with arguments from the literature that challenge the impartiality and consistency of Western powers, particularly with regard to their backing of Israel and their selective implementation of global norms^1,5,19^.

A substantial negative connection was identified between challenges and assessment, which was reduced by the adoption of Western values, when the researchers examined the intricate relationships among challenges, adoption, and assessment of Western values among Palestinian university students. This implies that the adoption of Western values functions as a mitigating factor^20^, minimizing the assessment-challenge negative association. The context for comprehending this dynamic is provided by the literature's emphasis on the U.S. and its allies' support for Israel and its role in suppressing human rights initiatives^5,6,14^.

The study revealed that demographic factors did not significantly impact the assessment and adoption of Western values among Palestinian university students. This emphasizes that the assessment and adoption of Western values among Palestinian university students are more intricately linked to global events and political dynamics than to individual characteristics^21^. Many Palestinians hold a positive view of the EU's peacebuilding model; however, there is significant disappointment with the EU's foreign policy behavior^4,6,14^.

This research revealed a significant gender disparity, with females scoring higher on the Perceived Difficulties of Western values (PcWV). This highlights the impact of gender on how people perceive and address Western values. This study underscores the significance of media bias and Western governments’ reactions to international crises, underscoring the critical role of gender dynamics in shaping perceptions^22^. The study revealed that respondents in urban and rural areas perceived more challenges related to Western values than did those in refugee camps, indicating media bias and differing experiences in interpreting Western values. This suggests that geographical differences may be due to specific narratives or unique experiences, influencing how people perceive these challenges. The study also underscores the multifaceted nature of challenges linked to Western values and their interpretation in diverse situations^22^, especially when we compare the War on Ukraine and the War on Gaza^1,4,8^.

Qualitatively, the data provide a detailed picture of how Palestinian students' views on Western ideals have changed over time, especially in the wake of the 2023 Gaza War. Many participants in the survey initially believed that Western values were superior and European countries hold a superior culture^4^ but the results showed a notable shift in attitudes. Nevertheless, there was a noticeable decline in faith in these ideals after the war, which emphasizes the need to understand these shifting attitudes.

The Gaza War of 2023 had a significant impact on the participants' outlooks, leading to an update of their experiences and beliefs. The observed decrease in belief in Western ideas was attributed to traumatic occurrences^3,20^ among Palestinians (e.g., the loss of 23 thousand people, the displacement of approximately two million people and the massive destruction of Gaza infrastructure), the impact of biased Western media on Israeli narratives^14^, and the contradiction between idealized standards and realistic reality. Biased Western media, as highlighted by^19^, played a role in shaping narratives favoring Israel, contributing to the observed contradiction between idealized standards and harsh realities^3,8,9^. This change may be linked to nationalistic feelings, notions of cultural and religious identity, and the belief that a particular group, i.e., Palestinians, is treated unjustly by Western norms^22^.

Palestinians' perceptions of Western values are influenced by a variety of factors, including geopolitical realities, perceptions of double standards, cultural and religious sensitivities, patriotic sentiments, global power dynamics, and experiences of isolation. The endorsement of policies detrimental to Palestinian interests by Western governments in the Israeli-Palestinian conflict, as pointed out by^3,8^, significantly shapes these perceptions. Israeli criticism can be vetoed or opposed by Western powers, especially the United States, which has recognized Jerusalem as Israel's capital and supported Israel militarily^19^. Media coverage has the potential to further mold negative sentiments, as stated by^1,3,14^, by casting doubt on the legitimacy of Western ideas, especially in regard to human rights.

Reluctance to accept or distance oneself from Western norms can be attributed to cultural and religious sensitivities^5^, such as identity preservation and worries about cultural appropriation^22^. Rooted in historical struggles for self-determination, nationalistic emotions see Western ideas as alien and therefore dangerous to Palestinian identity. The Palestinians have endured about seven decades of Israeli colonization^23^. Skepticism is further fostered by the selective application of Western values in international interactions^18^. It is further reinforced by exclusion from economic and aid initiatives, as well as from political and diplomatic spheres, that Western principles put the interests of the West first^1^. Israeli interest-driven economic development initiatives may exclude Palestinians, reinforcing that Western values place economic growth before Palestinian welfare^14,18^.

Some participants' awareness of and dedication to Arab and Islamic values have grown as a result of their view of a significant conflict between Arab-Islamic principles and Western ideals^24^. Conversely, some people think that Western principles are irrelevant. This contradiction is influenced by historical and geopolitical contexts when adhering to Arab-Islamic values is perceived as a defense against other forces, such as the 2023 war on Gaza^25^. The preservation of traditional Palestinian customs and heritage are seen as acts of resistance against perceived Western cultural intrusion, reflecting the belief that Western ideas are foreign to Palestinian identity^14^. Conflicts can encourage harmony and a sense of belonging to a shared cultural and religious past, but they can also spark a rejection of Western ideas that are seen as being forced^19^.

There was wide variance of opinion about Western intellectuals and influencers who support democracy and human rights^26^. While some found these figures attractive at first, others stopped following them after the Gaza war. Perceived double standards and social media dynamics all contribute to these diverse perspectives^1^. According to some participants, Western governments lacked the willingness and/or power-resources to compel Israel to peaceful coexistence^19^. Some people feel that Western governments support human rights causes in Ukraine but remain silent or take a different stance in the Israeli-Palestinian conflict^8^. According to many individuals, these positions have led to deeper feelings of nationalism and skepticism. These sentiments often arise from personal experiences of witnessing or living through the effects of the war^27^. As a result, some individuals have reevaluated their principles and allies, including local Palestinian factions^28,29^.

## Conclusion

This study explores the complex relationships among perceptions, challenges, and adoption of Western values among Palestinian university students. This study revealed a positive correlation between the perceived challenges of Western values and their assessment, aligning with the literature on geopolitical events such as the 2023 Gaza War. However, a negative link is found between the adoption of Western values and their assessment, suggesting that higher adoption rates diminish the inclination to appraise these values. The study also highlights the role of adoption as a mitigating factor, reducing the negative association between challenges and assessment. Although there is a notable gender and geographic difference in the assessment and adoption of Western values, females and those living in cities and villages are shown to have greater perceived challenges with these values.

This study provides a nuanced understanding of how Palestinian students' views on Western ideals evolved, particularly in the aftermath of the 2023 Gaza War. The research revealed a notable shift in attitudes, initially characterized by belief in the superiority of Western values, followed by a decline in faith after the war. This decline is attributed to traumatic events, biased media narratives, and the contradiction between idealized standards and harsh realities. This study also elucidates the multifaceted factors shaping Palestinians' perceptions of Western values, including geopolitical realities, double standards, cultural and religious sensitivities, experiences of isolation, media coverage, historical struggles, and selective application of Western values in international interactions.

In conclusion, the study emphasizes the need for a comprehensive understanding of the multifaceted influences on Palestinian perceptions of Western values.

## Strengths and weaknesses

This study has a number of strengths that add to its validity and reliability. Primarily, its all-encompassing methodology, which integrates both qualitative and quantitative techniques, permits an exhaustive investigation of the complex interplay between Palestinian college attendees and Western principles. By concentrating on the aftermath of the Gaza War in 2023, the research findings become more pertinent and contextually rich, and they have been placed at a pivotal point in recent history. Moreover, the incorporation of extant literature establishes a firm theoretical foundation for the research, thus strengthening the study's legitimacy. Remarkably, the discovery of a substantial gender gap broadens the scope of the investigation and highlights the complex ways in which gender shapes attitudes. This study, however, has certain drawbacks. The study may have limitations in terms of generalizability due to its concentration on Palestinian university students, and the use of self-reported data may induce biases. Moreover, the time limitations of the investigation impede a more thorough examination of the longitudinal development of perceptions, indicating potential areas for enhancement in subsequent research undertakings.

## Implications and future research

The findings of this study have significant implications for both academic discourse and practical considerations. A more nuanced understanding of how geopolitical events affect views is necessary, as seen by the positive link that has been found between Palestinian university students' judgment of Western ideals and perceived challenges to them. Policymakers, educators, and practitioners of international relations can all benefit from this insight, as they develop ways to recognize and address these dynamics. Furthermore, there is a negative association between adopting Western values and assessing them, which makes one wonder about the impartiality and consistency of Western powers and calls for a reassessment of diplomatic strategies.

Subsequent investigations may explore in greater detail the dynamic nature of these views after a conflict and their temporal evolution. Practical answers might come from investigating interventions or educational initiatives that try to close the gap between perceptions and actuality. Furthermore, broadening the demographic scope to encompass a range of socioeconomic backgrounds will enhance our understanding of how the different experiences of the Palestinian context influence attitudes toward Western values.

## Data Availability

The data that support the findings of this study are available from the corresponding author upon special request.
